# Transmission of *Mycobacterium tuberculosis* Beijing Strains, Alberta, Canada, 1991–2007

**DOI:** 10.3201/eid1905.121578

**Published:** 2013-05

**Authors:** Deanne Langlois-Klassen, Ambikaipakan Senthilselvan, Linda Chui, Dennis Kunimoto, L. Duncan Saunders, Dick Menzies, Richard Long

**Affiliations:** University of Alberta, Edmonton, Alberta, Canada (D. Langlois-Klassen, A. Senthilselvan, L. Chui, D. Kunimoto, L.D. Saunders, R. Long);; Provincial Laboratory for Public Health, Edmonton (L. Chui); McGill University Health Centre, Montreal, Quebec, Canada (D. Menzies);; McGill University, Montreal (D. Menzies)

**Keywords:** tuberculosis, epidemiology, disease transmission, infectious, emigrants and immigrants, Mycobacterium tuberculosis and other mycobacteria, bacteria, Canada

## Abstract

Transmission of Beijing strains posed no more of a public health threat than did non-Beijing strains.

## Medscape CME Activity

Medscape, LLC is pleased to provide online continuing medical education (CME) for this journal article, allowing clinicians the opportunity to earn CME credit. 

This activity has been planned and implemented in accordance with the Essential Areas and policies of the Accreditation Council for Continuing Medical Education through the joint sponsorship of Medscape, LLC and Emerging Infectious Diseases. Medscape, LLC is accredited by the ACCME to provide continuing medical education for physicians.

Medscape, LLC designates this Journal-based CME activity for a maximum of 1 *AMA PRA Category 1 Credit(s)*^TM^. Physicians should claim only the credit commensurate with the extent of their participation in the activity.

All other clinicians completing this activity will be issued a certificate of participation. To participate in this journal CME activity: (1) review the learning objectives and author disclosures; (2) study the education content; (3) take the post-test with a 70% minimum passing score and complete the evaluation at www.medscape.org/journal/eid; (4) view/print certificate.

**Release date: April 23, 2013; Expiration date: April 23, 2014**

## Learning Objectives

Upon completion of this activity, participants will be able to:

- Describe the epidemiology and previous research of the Beijing strains
- Distinguish characteristics of pulmonary TB with the Beijing strains in the current study
- Compare the transmissibility of the Beijing and non-Beijing strains in the current study
- Analyze factors associated with secondary cases of pulmonary TB in the current study

## CME Editor

**Karen L. Foster, **Technical Writer/Editor, Emerging Infectious Diseases. Disclosure: Karen L. Foster has disclosed no relevant financial relationships.

## CME Author

**Charles P. Vega, MD, **Health Sciences Clinical Professor; Residency Director, Department of Family Medicine, University of California, Irvine. *Disclosure: Charles P. Vega, MD, has disclosed no relevant financial relationships.*

## Authors

*Disclosures* ***: Deanne Langlois-Klassen, PhD; Ambikaipakan Senthilselvan, PhD; Linda Chui, PhD; Dennis Kunimoto, MD; L. Duncan Saunders, MBBCh, PhD; Dick Menzies, MD;*** *and* ***Richard Long, MD*** *, have disclosed no relevant financial relationships.*

## References

[1] European Concerted Action on New Generation Genetic Markers and Techniques for the Epidemiology and Control of Tuberculosis. Beijing genotype *Mycobacterium tuberculosis* and drug resistance. Emerg Infect Dis. 2006;12:736–43. 10.3201/eid1205.050400 16704829 PMC3374453

[2] Cowley D, Govender D, February B, Wolfe M, Steyn L, Evans J, Recent and rapid emergence of W-Beijing strains of *Mycobacterium tuberculosis* in Cape Town, South Africa. Clin Infect Dis. 2008;47:1252–9. 10.1086/592575 18834315

[3] Brudey K, Driscoll JR, Rigouts L, Prodinger WM, Gori A, Al-Hajoj SA, *Mycobacterium tuberculosis* complex genetic diversity: mining the fourth international spoligotyping database (SpoIDB4) for classification, population genetics and epidemiology. BMC Microbiol. 2006;6:23. 10.1186/1471-2180-6-23 16519816 PMC1468417

[4] Gallego B, Sintchenko V, Jelfs P, Coiera E, Gilbert GL. Three-year longitudinal study of genotypes of *Mycobacterium tuberculosis* in a low prevalence population. Pathology. 2010;42:267–72. 10.3109/00313021003631346 20350221

[5] Kato-Maeda M, Kim EY, Flores L, Jarlsberg LG, Osmond D, Hopewell PC. Differences among sublineages of the East-Asian lineage of *Mycobacterium tuberculosis* in genotypic clustering. Int J Tuberc Lung Dis. 2010;14:538–44. 20392345 PMC3625672

[6] Langlois-Klassen D, Kunimoto D, Saunders LD, Chui L, Boffa J, Menzies D, A population-based cohort study of *Mycobacterium tuberculosis* Beijing strains: an emerging public health threat in an immigrant-receiving country? PLoS ONE. 2012;7:e38431. 10.1371/journal.pone.0038431 22679504 PMC3367965

[7] Devaux I, Kremer K, Heersma H, Van Soolingen D. Clusters of multidrug-resistant *Mycobacterium tuberculosis* cases, Europe. Emerg Infect Dis. 2009;15:1052–60. 10.3201/eid1507.080994 19624920 PMC2744220

[8] Ghebremichael S, Groenheit R, Pennhag A, Koivula T, Andersson E, Bruchfeld J, Drug resistant *Mycobacterium tuberculosis* of the Beijing genotype does not spread in Sweden. PLoS ONE. 2010;5:e10893. 10.1371/journal.pone.0010893 20531942 PMC2878347

[9] López B, Aguilar D, Orozco H, Burger M, Espitia C, Ritacco V, A marked difference in pathogenesis and immune response induced by different *Mycobacterium tuberculosis* genotypes. Clin Exp Immunol. 2003;133:30–7. 10.1046/j.1365-2249.2003.02171.x 12823275 PMC1808750

[10] Manca C, Reed MB, Freeman S, Mathema B, Kreiswirth B, Barry CE III, Differential monocyte activation underlies strain-specific *Mycobacterium tuberculosis* pathogenesis. Infect Immun. 2004;72:5511–4. 10.1128/IAI.72.9.5511-5514.2004 15322056 PMC517425

[11] Toungoussova OS, Caugant DA, Sandven P, Mariandyshev AO, Bjune G. Impact of drug resistance on fitness of *Mycobacterium tuberculosis* strains of the W-Beijing genotype. FEMS Immunol Med Microbiol. 2004;42:281–90. 10.1016/j.femsim.2004.05.012 15477041

[12] Aguilar D, Hanekom M, Mata D, Gey van Pittius NC, van Helden PD, Warren RM, *Mycobacterium tuberculosis* strains with the Beijing genotype demonstrate variability in virulence associated with transmission. Tuberculosis (Edinb). 2010;90:319–25. 10.1016/j.tube.2010.08.004 20832364

[13] Theus S, Eisenach K, Fomukong N, Silver RF, Cave MD. Beijing family *Mycobacterium tuberculosis* strains differ in their intracellular growth in THP-1 macrophages. Int J Tuberc Lung Dis. 2007;11:1087–93. 17945065

[14] Coscolla M, Gagneux S. Does *M. tuberculosis* genomic diversity explain disease diversity? Drug Discov Today Dis Mech. 2010;7:e43–59. 10.1016/j.ddmec.2010.09.004 21076640 PMC2976975

[15] Borgdorff MW, Behr MA, Nagelkerke NJ, Hopewell PC, Small PM. Transmission of tuberculosis in San Francisco and its association with immigration and ethnicity. Int J Tuberc Lung Dis. 2000;4:287–94. 10777075

[16] World Health Organization. Global tuberculosis control: surveillance, planning, financing: WHO report 2008; 2008 [cited 2011 Apr 18]. http://www.who.int/tb/publications/global_report/2008/pdf/fullreport.pdf

[17] Public Health Agency of Canada, Canadian Lung Association/Canadian Thoracic Society. Canadian tuberculosis standards. 6th ed. Long R, Ellis E, eds. Ottawa (ON): Her Majesty the Queen in Right of Canada, represented by the Minister of Public Works and Government Services Canada; 2007.

[18] Kunimoto D, Sutherland K, Wooldrage K, Fanning A, Chui L, Manfreda J, Transmission characteristics of tuberculosis in the foreign-born and the Canadian-born populations of Alberta, Canada. Int J Tuberc Lung Dis. 2004;8:1213–20. 15527153

[19] Gagneux S, DeRiemer K, Van T, Kato-Maeda M, de Jong BC, Narayanan S, Variable host-pathogen compatibility in *Mycobacterium tuberculosis.* Proc Natl Acad Sci U S A. 2006;103:2869–73. 10.1073/pnas.0511240103 16477032 PMC1413851

[20] Tsolaki AG, Gagneux S, Pym AS, Goguet de la Salmoniere YO, Kreiswirth BN, Van Soolingen D, Genomic deletions classify the Beijing strains as a distinct genetic lineage of *Mycobacterium tuberculosis.* J Clin Microbiol. 2005;43:3185–91. 10.1128/JCM.43.7.3185-3191.2005 16000433 PMC1169157

[21] Borgdorff MW, van den Hof S, Kremer K, Verhagen L, Kalisvaart N, Erkens C, Progress towards tuberculosis elimination: secular trend, immigration and transmission. Eur Respir J. 2010;36:339–47. 10.1183/09031936.00155409 19996188

[22] Jasmer RM, Hahn JA, Small PM, Daley CL, Behr MA, Moss AR, A molecular epidemiologic analysis of tuberculosis trends in San Francisco, 1991–1997. Ann Intern Med. 1999;130:971–8. 10.7326/0003-4819-130-12-199906150-00004 10383367

[23] Hosmer DW, Lemeshow S. Applied logistic regression. 2nd ed. New York: Wiley; 2000.

[24] Frieden TR, Sherman LF, Maw KL, Fujiwara PI, Crawford JT, Nivin B, A multi-institutional outbreak of highly drug-resistant tuberculosis: epidemiology and clinical outcomes. JAMA. 1996;276:1229–35. 10.1001/jama.1996.03540150031027 8849750

[25] de Jong BC, Hill PC, Aiken A, Awine T, Antonio M, Adetifa IM, Progression to active tuberculosis, but not transmission, varies by *Mycobacterium tuberculosis* lineage in The Gambia. J Infect Dis. 2008;198:1037–43. 10.1086/591504 18702608 PMC2597014

[26] Marais BJ, Hesseling AC, Schaaf HS, Gie RP, Van Helden PD, Warren RM. *Mycobacterium tuberculosis* transmission is not related to household genotype in a setting of high endemicity. J Clin Microbiol. 2009;47:1338–43. 10.1128/JCM.02490-08 19261801 PMC2681880

[27] Nava-Aguilera E, Andersson N, Harris E, Mitchell S, Hamel C, Shea B, Risk factors associated with recent transmission of tuberculosis: systematic review and meta-analysis. Int J Tuberc Lung Dis. 2009;13:17–26. 19105874

[28] Lohmann EM, Koster BFPJ, le Cessie S, Kamst-van Agterveld MP, van Soolingen D, Arend SM. Grading of a positive sputum smear and the risk of *Mycobacterium tuberculosis* transmission. Int J Tuberc Lung Dis. 2012;16:1477–84. 10.5588/ijtld.12.0129 22964038

[29] Borgdorff MW, van Deutekom H, de Haas PE, Kremer K, van Soolingen D. *Mycobacterium tuberculosis,* Beijing genotype strains not associated with radiological presentation of pulmonary tuberculosis. Tuberculosis (Edinb). 2004;84:337–40. 10.1016/j.tube.2003.10.002 15207809

[30] Portevin D, Gagneux S, Comas I, Young D. Human macrophage responses to clinical isolates from the *Mycobacterium tuberculosis* complex discriminate between ancient and modern lineages. PLoS Pathog. 2011;7:e1001307. 10.1371/journal.ppat.1001307 21408618 PMC3048359

[31] Mokrousov I, Narvskaya O, Otten T, Vyazovaya A, Limeschenko E, Steklova L, Phylogenetic reconstruction within *Mycobacterium tuberculosis* Beijing genotype in northwestern Russia. Res Microbiol. 2002;153:629–37. 10.1016/S0923-2508(02)01374-8 12558181

[32] Tsao TCY, Hong J, Li LF, Hsieh MJ, Liao SK, Chang KSS. Imbalances between tumor necrosis factor-alpha and its soluble receptor forms, and interleukin-1 beta and interleukin-1 receptor antagonist in BAL fluid of cavitary pulmonary tuberculosis. Chest. 2000;117:103–9. 10.1378/chest.117.1.103 10631206

[33] Kremer K, Glynn JR, Lillebaek T, Niemann S, Kurepina NE, Kreiswirth BN, Definition of the Beijing/W lineage of *Mycobacterium tuberculosis* on the basis of genetic markers. J Clin Microbiol. 2004;42:4040–9. 10.1128/JCM.42.9.4040-4049.2004 15364987 PMC516354

[34] Langlois-Klassen D, Wooldrage K, Manfreda J, Sutherland K, Ellis E, Phypers M, Piecing the puzzle together: foreign-born tuberculosis in an immigrant-receiving country. Eur Respir J. 2011;38:895–902. 10.1183/09031936.00196610 21436350

[35] Vanhomwegen J, Kwara A, Martin M, Gillani FS, Fontanet A, Mutungi P, Impact of immigration on the molecular epidemiology of tuberculosis in Rhode Island. J Clin Microbiol. 2011;49:834–44. 10.1128/JCM.01952-10 21159930 PMC3067685

[36] Smieja MJ, Marchetti CA, Cook DJ, Smaill FM. Isoniazid for preventing tuberculosis in non-HIV infected persons. Cochrane Database Syst Rev. 2000; (2):CD001363. 10796642 10.1002/14651858.CD001363 PMC6532737

[37] Schwartzman K, Oxlade O, Barr RG, Grimard F, Acosta I, Baez J, Domestic returns from investment in the control of tuberculosis in other countries. N Engl J Med. 2005;353:1008–20. 10.1056/NEJMsa043194 16148286

[38] Glynn JR, Bauer J, de Boer AS, Borgdorff MW, Fine PE, Godfrey-Faussett P, European Concerted Action on Molecular Epidemiology and Control of Tuberculosis. Interpreting DNA fingerprint clusters of *Mycobacterium tuberculosis.* Int J Tuberc Lung Dis. 1999;3:1055–60. 10599007

[39] Tanaka MM, Phong R, Francis AR. An evaluation of indices for quantifying tuberculosis transmission using genotypes of pathogen isolates. BMC Infect Dis. 2006;6:92. 10.1186/1471-2334-6-92 16756684 PMC1538606

[40] Jensen M, Lau A, Langlois-Klassen D, Boffa J, Manfreda J, Long R. A population-based study of tuberculosis epidemiology and innovative service delivery in Canada. Int J Tuberc Lung Dis. 2012;16:43–9. 10.5588/ijtld.11.0374 22236844

