# Supplementary material for: Transmission of Mycobacterium tuberculosis Beijing Strains, Alberta, Canada, 1991–2007
Source: Emerg Infect Dis. 2013 May;19(5):701–11. doi: 10.3201/eid1905.121578 (PMC3649004; doi:10.3201/eid1905.121578)
Supplement: Technical Appendix — Kaplan-Meier estimates of the probability of an identical DNA fingerprint pattern match in respiratory tuberculosis cases in Alberta, Canada, 1991–2007. [file 12-1578-Techapp-s1.pdf]

Article DOI: http://dx.doi.org/10.3201/eid1905.121578

Transmission of *Mycobacterium tuberculosis* Beijing Strains, Alberta, Canada, 1991–2007

Technical Appendix

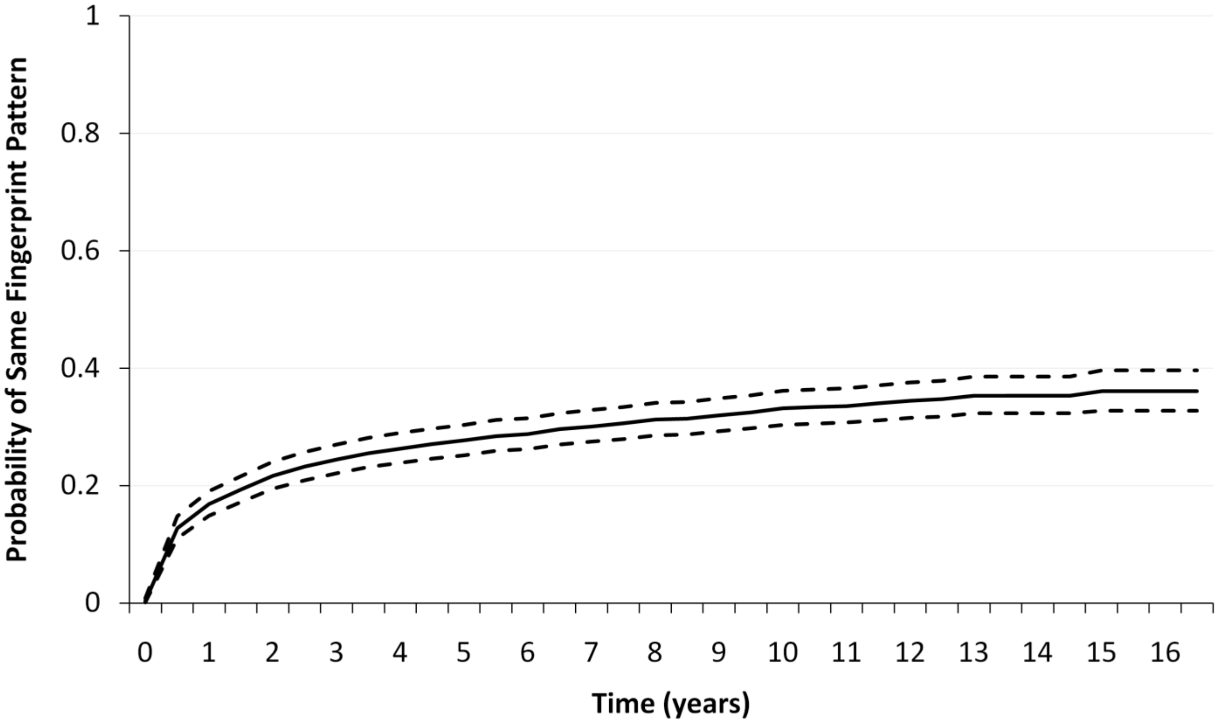

Technical Appendix Figure. Kaplan-Meier estimates of the probability of an identical DNA fingerprint pattern match in respiratory TB cases in Alberta, Canada, 1991–2007. The estimated probabilities (solid line) and 95% CIs (dashed lines) were derived through a Kaplan-Meier analysis of the time between the dates of diagnosis of cases with identical DNA fingerprint patterns of IS*6110* restriction fragment length polymorphism analysis (as well as spoligotyping for isolates with $\leq$5 copies of IS*6110*). Cases with unique DNA fingerprint patterns (i.e., no other case had an identical DNA fingerprint pattern during 1991–2007) were censored at the end of the study period (June 30, 2007).
