# Supplementary material for: Transmission of Mycobacterium tuberculosis Beijing Strains, Alberta, Canada, 1991–2007
Source: Emerg Infect Dis. 2013 May;19(5):701–11. doi: 10.3201/eid1905.121578 (PMC3649004; doi:10.3201/eid1905.121578)
Supplement: Related texts

# Research article: Transmission of *Mycobacterium tuberculosis* Beijing Strains, Alberta, Canada, 1991–2007

**Authors:** Deanne Langlois-Klassen, Ambikaipakan Senthilselvan, Linda Chui, Dennis Kunimoto, L. Duncan Saunders, Dick Menzies, Richard Long

**Affiliations:** University of Alberta, Edmonton, Alberta, Canada (D. Langlois-Klassen, A. Senthilselvan, L. Chui, D. Kunimoto, L.D. Saunders, R. Long);; Provincial Laboratory for Public Health, Edmonton (L. Chui); McGill University Health Centre, Montreal, Quebec, Canada (D. Menzies);; McGill University, Montreal (D. Menzies)

## Abstract

Beijing strains are speculated to have a selective advantage over other *Mycobacterium tuberculosis* strains because of increased transmissibility and virulence. In Alberta, a province of Canada that receives a large number of immigrants, we conducted a population-based study to determine whether Beijing strains were associated with increased transmission leading to disease compared with non-Beijing strains. Beijing strains accounted for 258 (19%) of 1,379 pulmonary tuberculosis cases in 1991–2007; overall, 21% of Beijing cases and 37% of non-Beijing cases were associated with transmission clusters. Beijing index cases had significantly fewer secondary cases within 2 years than did non-Beijing cases, but this difference disappeared after adjustment for demographic characteristics, infectiousness, and *M. tuberculosis* lineage. In a province that has effective tuberculosis control, transmission of Beijing strains posed no more of a public health threat than did non-Beijing strains.

The Beijing lineage of *Mycobacterium tuberculosis* (also referred to as the East Asian lineage or lineage 2) is an emerging public health threat (*1*,*2*). The Beijing lineage accounts for 13% of *M. tuberculosis* strains globally (*3*) and 19%–27% of *M. tuberculosis* strains in low tuberculosis (TB) incidence immigrant-receiving countries, such as Australia, the United States, and Canada (*4*–*6*). In addition to their recent global dissemination, Beijing lineage strains raise concern because of frequent associations with drug resistance and multidrug-resistant TB in particular (*1*,*6*–*8*). Reports of Beijing strains that are extensively drug resistant further intensify these concerns (*7*).

The rapid global expansion of Beijing strains and their frequent (albeit inconsistent) association with large TB outbreaks and younger patients has led to speculation that Beijing strains have a selective advantage over other *M. tuberculosis* lineages as conferred through increased transmissibility and virulence (*1*,*2*,*7*). This hypothesis is supported by experimental evidence of the increased virulence of Beijing lineage strains relative to other *M. tuberculosis* strains in vitro and in animal models (*9*,*10*). Evidence also suggests that the fitness of some Beijing strains is retained after the acquisition of drug resistance (*11*). Nevertheless, intragenotypic variation in virulence has been described in the Beijing family (*12*,*13*) and, in a review, Coscolla and Gagneux (*14*) concluded that the current body of evidence is insufficient to support the increased transmissibility of these strains.

Immigration is the main determinant of TB epidemiology in low incidence settings (*15*,*16*). Consequently, the importation of potentially more pathogenic strains, such as those in the Beijing family, could have major implications for TB prevention and elimination efforts within immigrant-receiving countries. Surveillance activities that identify the sources and transmission patterns of emerging and/or expanding *M. tuberculosis* strains will be increasingly vital if TB prevention and care programs are to maintain their effectiveness within the context of dynamic immigration policies and highly mobile populations.

We aimed to investigate the association of Beijing and non-Beijing lineage strains with transmission in a low TB incidence immigrant-receiving province of Canada. In particular, we sought to determine whether the Beijing lineage of *M. tuberculosis* is a greater public health threat than other strains because of increased transmission leading to disease.

## Methods

### Study Setting and Population

Culture-confirmed pulmonary TB cases (potential transmitters) in the province of Alberta, Canada, during January 1, 1991–June 30, 2007 (i.e., study period) in accordance with the provincial TB registry were eligible for inclusion in this population-based retrospective study (see Transmission Leading to Disease below for additional criteria). These cases represent the pulmonary subset of previously reported cases (*6*). Ethics approval was received from the University of Alberta Health Research Ethics Board, and analysis of surveillance data did not require informed consent because there was no direct patient contact.

Persons born in Canada or born outside of Canada to Canadian-born parents were considered Canadian-born; all others were foreign-born. The Canadian-born population was not further categorized into Aboriginal and non-Aboriginal groups because only 5 Beijing TB cases occurred among Aboriginal peoples during the study period (*6*). However, because of the high prevalence of Beijing strains in parts of Southeast and East Asia (*1*), country of birth was used to group foreign-born persons into those born in the Western Pacific region and those born elsewhere (*16*).

### Case Characteristics

Demographic and clinical data from the TB Registry were combined with data from the Provincial Laboratory for Public Health (Provincial Laboratory). The Provincial Laboratory conducts all mycobacteriology studies in the province in accordance with the Canadian Tuberculosis Standards (*17*).

Sputum smear status and the presence or absence of lung cavitation on chest radiograph were used as indicators of infectiousness. Baseline sputum smears collected on, or within the week before, the date of diagnosis (the start date of treatment) that had grade 3+ to 4+ scores for acid-fast bacilli were categorized as having high bacillary loads (*17*). Monoresistant isolates had resistance to a single first-line drug, namely isoniazid, rifampin, pyrazinamide, ethambutol, or streptomycin (*17*). Resistance to >2 first-line drugs but without isoniazid–rifampin resistance constituted polyresistance, whereas multidrug-resistant TB comprised cases with resistance to at least isoniazid–rifampin.

The Provincial Laboratory completed DNA fingerprinting of prospectively archived isolates with the IS*6110* restriction fragment-length polymorphism (RFLP) method, and for isolates with <5 copies of IS*6110*, spoligotyping was performed as described (*18*). Isolates were also assigned to an *M. tuberculosis* lineage at the Provincial Laboratory according to the PCR-based detection of large sequence polymorphisms as described (*19*,*20*). Isolates with a deletion of RD105 were classified as Beijing lineage strains and all others as non-Beijing lineage strains.

### Transmission Leading to Disease

Of the 1,399 eligible pulmonary TB cases during the study period, 20 (1%) were excluded because either the DNA fingerprint pattern or *M. tuberculosis* lineage could not be determined. The remaining 1,379 cases were included in an analysis of clustering to provide an indication of overall transmission leading to disease during the study period. A cluster was defined as >2 patients whose case isolates had identical DNA fingerprints.

In addition to overall transmission, recent transmission that led to disease was quantified to account for dissimilarities in the follow-up periods of potential source cases and to minimize the probability of propagated transmission by second and later generation source cases (*21*). A Kaplan-Meier survival analysis was completed with the 1,379 pulmonary TB cases to determine the cutoff point for the definition of recent transmission (*21*,*22*). The Kaplan-Meier probability of an isolate being followed by another isolate with an identical fingerprint pattern during the 16.5-year study period was 0.36; the probability of >2 isolates having identical fingerprint patterns in a 2- and 3-year period was 0.22 and 0.24, respectively (online Technical Appendix, wwwnc.cdc.gov/EID/article/19/5/12-1578-Techapp1.pdf). Given the similarity in these latter probabilities, the 2-year period was subsequently determined to be the ideal cutoff period because it coincided with the conventional high-risk period for the development of active TB after recent infection (18–24 months) (*17*).

Using the 2-year cutoff point, we defined an index case as a pulmonary TB case with a DNA fingerprint pattern that had not been assigned to another case within the preceding 2 years. A secondary case was any case that had an identical fingerprint pattern as an index case provided that it was also diagnosed no more than 2 years after the index case. Using these definitions, we excluded 430 (31%) of 1,379 TB cases from the analysis of recent transmission. Specifically, 167 index cases diagnosed during 1991–1992 and their 50 secondary cases were excluded because we could not determine whether the fingerprint patterns of the index cases matched another case in the preceding 2 years. Follow-up periods of <2 years resulted in the exclusion of an additional 124 index cases diagnosed after June 2005 and their 10 secondary cases. Finally, 79 secondary cases were excluded because of diagnosis >2 years after the index case but <2 years after another cluster member. After these exclusions, 949 (69%) cases diagnosed during January 1, 1993–June 30, 2007, were included in the primary analysis of recent transmission.

### Statistical Analysis

We analyzed data using Stata/IC 11 (StataCorp LP, College Station, TX, USA). For overall transmission, associations between case characteristics and *M. tuberculosis* lineage were assessed with bivariate and multivariate logistic regression analyses at a 5% level of significance. Characteristics of case-patients (sex, age at diagnosis, population group, sputum smear status, bacillary load, lung cavitation, drug resistance and clustering) that were p<0.2 in bivariate analyses were eligible for inclusion in the multivariate model. Subgroup analyses were also completed to evaluate intragenotypic associations between clustering and case-patient characteristics by using bivariate and multivariate logistic regression analyses.

For the analyses of recent transmission, transmission indices were calculated as the total number of secondary cases within the cutoff period divided by the total number of index cases (*21*). The risk factors of index cases that were associated with recent transmission leading to disease (i.e., relative transmission indices) were assessed with bivariate and multivariate Poisson regression by using an offset of 1 for each index case (*21*). Specifically, associations with sex, age at diagnosis, population group, sputum smear status, bacillary load, lung cavitation, drug resistance, and *M. tuberculosis* lineage were initially analyzed with bivariate Poisson regression. Multivariate Poisson regression modeling was constructed with purposeful selection and, with the exception of *M. tuberculosis* lineage, variables that had p<0.20 in bivariate regression were included in the initial multivariate model. As the primary variable of interest in this study, *M. tuberculosis* lineage was included in all multivariate models regardless of its significance.

For all multivariate regression modeling, the confounding effects of removed variables (p>0.05) were assessed with the percentage rule. We used a collapsibility criterion <15%, and the significance of potential interactions was based on the partial likelihood ratio test (*23*).

We assessed the influence of the length of the cutoff period on recent transmission with sensitivity analyses using 3- and 5-year cutoff periods. Additional analysis was completed with no cutoff period, the index case for each fingerprint pattern being the isolate in the dataset with the earliest date of diagnosis. We also evaluated the potential effect of including nonpulmonary secondary cases in the analysis of recent transmission. For this latter analysis, all nonpulmonary TB cases registered in Alberta during the study period that had an identical DNA fingerprint as a pulmonary index case were eligible for study inclusion.

## Results

We identified Beijing strains in 258 (19%) of the 1,379 pulmonary TB cases in 1991–2007. Compared with non-Beijing cases, Beijing cases occurred among persons of similar sex and age but more often foreign-born (p<0.0001) (Table 1). The infectiousness of Beijing and non-Beijing cases was similar in relation to sputum smear status, bacillary load, and presence/absence of lung cavitation (Table 1). *M. tuberculosis* lineage and drug resistance were not independently associated (Table 1).

**Table 1:** Characteristics of persons with pulmonary TB associated with *Mycobacterium tuberculosis* Beijing and non-Beijing strains, Alberta, Canada, 1991–2007

Characteristic	Strain			OR (95% CI)*	
	Beijing, no. (%)	Non-Beijing, no. (%)		Unadjusted	Adjusted
Sex					
F	100 (38.8)	492 (43.9)		1.0	1.0
M	158 (61.2)	629 (56.1)		1.2 (0.9–1.6)	1.3 (0.9–1.8)
Age at diagnosis, y					
<35	72 (27.9)	319 (28.5)		1.0	1.0
35–64	69 (26.7)	421 (37.6)		0.7 (0.5–1.04)	0.7 (0.1–1.01)
>64	117 (45.3)	381 (34.0)		1.4 (0.98–1.9)	1.1 (0.7–1.6)
Population group					
Canadian-born	19 (7.4)	520 (46.4)		1.0	1.0
Foreign-born, other	26 (10.1)	346 (30.9)		**2.1 (1.1–3.8)**	**2.1 (1.1–4.1)**
Foreign-born, Western Pacific	213 (82.6)	255 (22.7)		**22.9 (14.0–37.4)**	**22.6 (13.1–39.2)**
Sputum smear status†					
Negative	132 (54.1)	517 (49.0)		1.0	1.0
Positive	112 (45.9)	538 (51.0)		0.8 (0.6–1.1)	1.2 (0.8–1.7)
Bacillary load‡§					
Low	38 (70.4)	177 (66.5)		1.0	
High	16 (29.6)	89 (33.5)		0.8 (0.4–1.6)	
Results of chest radiography					
No cavitation	206 (79.8)	853 (76.1)		1.0	1.0
Cavitation	52 (20.2)	268 (23.9)		0.8 (0.6–1.1)	0.8 (0.5–1.3)
Drug resistance					
Pan-susceptible	202 (78.3)	1000 (89.2)		1.0	1.0
Monoresistance	30 (11.6)	90 (8.0)		**1.7 (1.1–2.6)**	0.8 (0.5–1.3)
Polyresistance	20 (7.8)	25 (2.2)		**4.0 (2.2–7.3)**	1.7 (0.9–3.5)
Multidrug resistance	6 (2.3)	6 (0.5)		**5.0 (1.6–15.5)**	3.7 (0.9–14.7)
Clustering					
Nonclustered	203 (78.7)	703 (62.7)		1.0	1.0
Clustered	55 (21.3)	418 (37.3)		**0.5 (0.3–0.6)**	0.8 (0.6–1.3)
Total	258 (100.0)	1,121 (100.0)			

*Boldface indicates significance (p<0.05). TB, tuberculosis; OR, odds ratio.  †Sputum smear microscopy was not completed for all cases. ‡Semiquantitative scores for acid-fast bacilli load on the baseline sputum smear. Positive smears with semiquantitative scores of 3+ or 4+ were categorized as having high bacillary load; all remaining positive smears were labeled as having a low bacillary load. §Bacillary load was not included in multivariate modeling because of multicollinearity with sputum smear status.

### Overall Transmission

Overall, 906 (66%) cases exhibited unique fingerprint patterns (nonclustered cases), and 473 (34%) clustered cases were dispersed among 119 clusters. Of Beijing cases, 203 (79%) were nonclustered, and 55 (21%) were distributed among 22 clusters. Non-Beijing cases accounted for the remaining 703 (63%) nonclustered cases and 418 (37%) clustered cases within 97 clusters. Beijing cases were half as likely as non-Beijing cases to be clustered (p<0.0001), but this difference disappeared after we controlled for demographic characteristics, infectiousness, and drug resistance (p = 0.405) (Table 1).

Intragenotypic analysis showed that the clustering of Beijing cases was not associated with demographic characteristics, infectiousness, or drug resistance (Table 2). In contrast, the likelihood of non-Beijing cases being clustered was significantly less when patients were >64 years of age at diagnosis (vs. <35 years; p<0.0001) or foreign-born (p<0.0001) (Table 2). Although resistance to a single first-line anti-TB drug appeared to be associated with less clustering in bivariate analysis (p = 0.001), it was not associated with clustering independent of sex, age at diagnosis, and population group (p = 0.102) (Table 2).

**Table 2:** Case-patient characteristics associated with clustering of Beijing and non-Beijing *Mycobacterium tuberculosis* lineages, Alberta, Canada, 1991–2007

Characteristic	All cases	No. (%) clustered cases	OR (95% CI)*	
			Unadjusted	Adjusted†
Beijing				
Sex				
F	100	19 (19.0)	1.0	
M	158	36 (22.8)	1.3 (0.7–2.3)	
Age at diagnosis, y				
<35	72	17 (23.6)	1.0	1.0
35–64	69	19 (27.5)	1.2 (0.6–2.6)	1.3 (0.6–2.8)
>64	117	19 (16.2)	0.6 (0.3–1.3)	0.7 (0.3–1.5)
Population group				
Canadian-born	19	7 (36.8)	1.0	
Foreign-born, other	26	6 (23.1)	0.5 (0.1–1.9)	
Foreign-born, Western Pacific	213	42 (19.7)	0.4 (0.2–1.1)	
Sputum smear status				
Negative	132	26 (19.7)	1.0	
Positive	112	25 (22.3)	1.2 (0.6–2.2)	
Bacillary load				
Low	38	7 (18.4)	1.0	
High	16	6 (37.5)	2.7 (0.7–9.8)	
Chest radiography				
No cavitation	206	40 (19.4)	1.0	1.0
Cavitation	52	15 (28.8)	1.7 (0.8–3.4)	1.6 (0.8–3.2)
Drug resistance				
Pan-susceptible	202	42 (20.8)	1.0	
Monoresistance	30	7 (23.3)	1.2 (0.5–2.9)	
Polyresistance	20	6 (30.0)	1.6 (0.6–4.5)	
Multidrug resistance	6	0	NA	
Non-Beijing				
Sex				
F	492	168 (34.1)	1.0	1.0
M	629	250 (39.7)	1.3 (0.98–1.6)	1.1 (0.8, 1.4)
Age at diagnosis, y				
<35	319	134 (42.0)	1.0	1.0
35–64	421	182 (43.2)	1.1 (0.8–1.4)	0.8 (0.5–1.1)
>64	381	102 (26.8)	**0.5 (0.4–0.7)**	**0.4 (0.3–0.6)**
Population group				
Canadian-born	520	316 (60.8)	1.0	1.0
Foreign-born, other	346	42 (12.1)	**0. 1 (0.06–0.13)**	**0.09 (0.06–0.13)**
Foreign-born, Western Pacific	255	60 (23.5)	**0.20 (0.14–0.28)**	**0.21 (0.15–0.31)**
Sputum smear status				
Negative	517	198 (38.3)	1.0	
Positive	538	199 (37.0)	0.9 (0.7–1.2)	
Bacillary load				
Low	177	67 (37.9)	1.0	
High	89	39 (43.8)	1.3 (0.8–2.1)	
Chest radiography				
No cavitation	853	312 (36.6)	1.0	
Cavitation	268	106 (39.6)	1.1 (0.9–1.5)	
Drug resistance				
Pan-susceptible	1,000	393 (39.3)	1.0	1.0
Monoresistance	90	19 (21.1)	**0.4 (0.2–0.7)**	0.6 (0.4–1.1)
Polyresistance	25	5 (20.0)	0.4 (0.1–1.04)	0.7 (0.3–2.1)
Multidrug resistance	6	1 (16.7)	0.3 (0.04–2.7)	0.5 (0.05–5.0)

*Boldface indicates significance (p<0.05). OR, odds ratio.  †Multivariate analysis was based on purposeful selection with variables with p<0.2 in unadjusted analyses being included in the multivariate model. For Beijing strains, multivariate analysis included the independent variables of age at diagnosis and chest radiography. Sex, age at diagnosis, population group, and drug resistance were the independent variables in the multivariate analysis for non-Beijing strains.

In each lineage group, the number of nonclustered TB cases in foreign-born persons was inversely associated with time since arrival, such that 30%–32% of these cases occurred within the first 2 years after arrival (Figure). Clustered cases appeared to follow a similar pattern (Figure), although interpretation was limited by the relatively small number of cases.

**Figure:** Number of clustered and nonclustered cases according to *Mycobacterium tuberculosis* lineage among foreign-born persons and time since arrival in Alberta, Canada, 1991–2007. A) Beijing cases; B) Non-Beijing cases. Gray bars, nonclustered cases; black bars, clustered cases.

### Recent Transmission

Cases excluded from the analysis of recent transmission were demographically and clinically similar to included cases (data not shown). On average, an index case resulted in 0.13 secondary cases within 2 years. In unadjusted analysis, the number of secondary cases was higher if the index case-patient was sputum smear positive, had a high bacillary load, or had lung cavitation (Table 3). Conversely, fewer secondary cases were associated with index case-patients who were >64 years of age (vs. <35 years), foreign-born, or infected with Beijing strains. In adjusted analysis, the number of secondary cases was associated with the age, population group, and smear status of the index case-patients (Table 3). Specifically, fewer secondary cases occurred if the index case-patient was >64 years of age (vs. <35 years) or foreign-born whereas an increased number of secondary cases was associated with sputum smear–positive index case-patients (Table 3). The lineage of *M. tuberculosis* was not independently associated with the number of secondary cases.

**Table 3:** Risk factors for the recent transmission of *Mycobacterium tuberculosis*, Alberta, Canada, 1993–2007

Characteristic	Index case-patient		Secondary case-patient	Transmission index*	Relative transmission index (95% CI)†	
	Nonclustered	Clustered			Unadjusted	Adjusted
Sex						
F	330	29	38	0.11	1.0	
M	440	44	68	0.14	1.3 (0.9–2.0)	
Age at diagnosis, y						
<35	185	17	30	0.15	1.0	1.0
35–64	277	35	47	0.15	1.0 (0.6–1.6)	0.6 (0.4–1.0)‡
>64	308	21	29	0.09	**0.6 (0.4–1.0)**§	**0.4 (0.3–0.7)**
Population group						
Canadian-born	210	46	77	0.30	1.0	1.0
Foreign-born, other	243	9	9	0.04	**0.1 (0.1–0.2)**	**0.1 (0.1–0.2)**
Foreign-born, Western Pacific	317	18	20	0.06	**0.2 (0.1–0.3)**	**0.2 (0.1–0.3)**
Sputum smear status§						
Negative	364	25	36	0.09	1.0	1.0
Positive	357	46	68	0.17	**1.8 (1.2–2.7)**	**1.6 (1.0–2.3)¶**
Bacillary load#						
Low	134	15	25	0.17	1.0	
High	56	15	23	0.32	**1.9 (1.1–3.4)**	
Chest radiography						
No cavitation	596	44	65	0.10	1.0	
Cavitation	174	29	41	0.20	**2.0 (1.3–2.9)**	
Drug resistance						
Pan-susceptible	660	66	98	0.13	1.0	
Monoresistance	72	4	4	0.05	0.4 (0.1–1.1)	
Polyresistance	28	3	4	0.13	1.0 (0.4–2.6)	
Multidrug resistance**	10	0	0	0.00		
*M. tuberculosis* strain						
Non-Beijing	597	61	94	0.14	1.0	0.8 (0.4–1.7)
Beijing	173	12	12	0.06	**0.5 (0.2–0.8)**	
Total	770	73	106	0.13		

*The number of secondary cases divided by the number of index cases †Bivariate and multivariate Poisson regression models used an offset of 1 each index case. Variables with p<0.20 in bivariate analysis were eligible for inclusion in the multivariate model.** Boldface** indicates significance (p<0.05). ‡p = 0.058. §Sputum smear microscopy was not completed for all cases. ¶p = 0.037. #Bacillary load was not included in multivariate modeling because of multicollinearity with sputum smear status. **Multidrug-resistant TB was excluded from bivariate and multivariate analyses.

In subgroup analyses of foreign-born index case-patients, the number of secondary cases per index case-patient was unrelated to the length of residency in Canada. For example, compared with index case-patients who were <2 years since arrival, the relative transmission indices of those 3–5 years and those >20 years since arrival were 1.1 (95% CI 0.3–4.1) and 1.2 (95% CI 0.4–3.6), respectively. Among persons born in the Western Pacific, no risk factors for the number of secondary cases within 2 years per index case-patient were identified, including age, *M. tuberculosis* lineage, or time since arrival (Table 4).

**Table 4:** Risk factors for the recent transmission of *Mycobacterium tuberculosis* among index case-patients born in Alberta, Canada, in the Western Pacific, 1993–2007

Characteristic	Index case-patients		Secondary case-patients	Transmission index*	Relative transmission index (95% CI)†	
	Nonclustered	Clustered			Unadjusted	Adjusted
Sex						
F	122	9	10	0.08	1.0	
M	195	9	10	0.05	0.6 (0.3–1.5)	
Age at diagnosis, y						
<35	83	5	5	0.06	1.0	
35–64	110	4	4	0.04	0.6 (0.2–2.3)	
>64	124	9	11	0.08	1.5 (0.5–4.2)	
Sputum smear status‡						
Negative	174	7	7	0.04	1.0	1.0
Positive	126	9	11	0.08	2.1 (0.8–5.4)	2.1 (0.8–5.5)
Bacillary load§						
Low	53	3	5	0.09	1.0	
High	17	1	1	0.06	0.6 (0.1–5.3)	
Chest radiography						
No cavitation	252	14	16	0.06	1.0	
Cavitation	65	4	4	0.06	1.0 (0.3–2.9)	
Drug resistance						
Pan-susceptible	239	13	14	0.06	1.0	
Monoresistance	51	2	2	0.04	0.7 (0.2–3.0)	
Polyresistance	21	3	4	0.17	3.0 (1.0–9.1)	
Multidrug resistance¶	6	0	0	0		
*M. tuberculosis* lineage						
Non-Beijing	171	9	11	0.06	1.0	1.0
Beijing	146	9	9	0.06	1.0 (0.4–2.3)	0.9 (0.3–2.2)
Time since arrival in Canada, y						
<2	76	3	3	0.04	1.0	
3–5	43	1	1	0.02	0.6 (0.1–5.8)	
6–10	53	6	6	0.10	2.7 (0.7–10.7)	
>10	112	8	10	0.08	2.2 (0.6–8.0)	
Total	317	18	20	0.06		

*The number of secondary cases divided by the number of index cases. †Bivariate Poisson regression using an offset of 1 each index case. ‡Sputum smear microscopy was not completed with all cases. §Bacillary load was not included in multivariate modeling because of multicollinearity with sputum smear status. ¶Multidrug-resistant tuberculosis was excluded from bivariate analyses.

Sensitivity analyses demonstrated that longer cutoff periods produced higher transmission indices among Beijing cases and non-Beijing cases (Table 5). Although Beijing index cases had significantly fewer secondary cases than did non-Beijing index cases regardless of the length of the cutoff period, *M. tuberculosis* lineage was not associated with the number of secondary cases within a 2-, 3-, or 5-year cutoff period after we controlled for demographic characteristics and infectiousness. Beijing index cases had significantly fewer secondary cases than did non-Beijing cases independent of other factors when a cutoff period was not defined (equivalent to overall transmission). Inclusion of nonpulmonary secondary cases also increased the transmission indices of Beijing and non-Beijing strains but had no significant effect on relative transmission indices.

**Table 5:** Sensitivity analyses of the relative transmission index of *Mycobacterium tuberculosis* lineage, Alberta, Canada, 1991–2007

Variable	Transmission Index			Relative transmission index (95% CI)*	
	Beijing	Non-Beijing		Unadjusted	Adjusted†
Cutoff period, y					
2	0.06	0.14		**0.45 (0.25–0.83)**	0.82 (0.41–1.67)
3	0.09	0.18		**0.50 (0.29–0.89)**	1.04 (0.53–2.06)
5	0.11	0.33		**0.33 (0.17–0.63)**	0.86 (0.41–1.79)
Overall	0.15	0.39		**0.38 (0.26–0.54)**	**0.58 (0.39–0.87)**
Inclusion of nonpulmonary secondary cases‡	0.07	0.17		**0.42 (0.23–0.74)**	0.70 (0.36–1.36)

*Boldface indicates significance (p<0.05). †Adjusted for sex, age at diagnosis, population group, sputum smear status, lung cavitation and *M. tuberculosis* lineage. ‡Using a 2-year cutoff period.

## Discussion

Outbreaks of *M. tuberculosis* Beijing lineage strains in high and low TB incidence settings have had major public health implications (*7*,*24*). Notwithstanding the effect of these outbreaks, we found the transmission of Beijing strains to be similar to that of non-Beijing strains in Alberta, a low TB incidence immigrant-receiving province of Canada. Speculation about the increased transmissibility of Beijing strains also has been refuted in other low incidence immigrant-receiving countries and in The Gambia (*8*,*25*). In South Africa, findings about the transmissibility of Beijing strains have been conflicting (*2*,*26*). The general absence of evidence to suggest that Beijing strains are inherently more transmissible than other *M. tuberculosis* lineages is highly informative for TB prevention and care programs, given the propensity for multidrug-resistant TB among persons infected with Beijing strains (*6*–*8*).

*M. tuberculosis* is transmitted most frequently when persons with TB have positive sputum acid-fast bacilli results, especially positive results with higher semiquantitative grades, and lung cavitation (*27*,*28*). Consequently, previous findings that Beijing strains are not typically associated with sputum smear positive or cavitary disease (*6*,*29*) accords with reported similarities in the transmission of Beijing and non-Beijing strains. In our study, the infectiousness of Beijing and non-Beijing cases was similar in terms of sputum smear positivity and bacillary load, whereas the likelihood of cavitation was significantly less for Beijing cases.

That Beijing strains have been associated with increased transmission in some settings may reflect geographic variations in virulence phenotypes. In the *M. tuberculosis* complex, evolutionarily modern lineages (including the Beijing lineage) induce weaker immune responses than do ancient lineages, and this response possibly provides modern lineages with a selective advantage in terms of more rapid disease progression and transmission in the human population (*30*). An array of virulence phenotypes also have been demonstrated in the more evolutionarily recent subfamily of Beijing strains (i.e., the modern subfamily as characterized by the insertion of IS*6110* in the noise transfer function chromosomal region [*31*]), including differences in the pathogenic characteristics (and potential transmissibility) of closely related strains in the same sublineage (*12*,*13*). For example, strains in the modern Beijing subfamily have significant variations in their intracellular growth rates and hence significant differences in tumor necrosis factor-α levels (*13*). This variation may be of particular relevance because of higher tumor necrosis factor-α levels in the bronchoalveolar lavage fluid of TB patients with large cavities (*32*).

To better understand the potential implications of virulence phenotypes, it would be of benefit if future population-based investigations in high and low incidence settings discerned between the disease characteristics and transmissibility of different *M. tuberculosis* subfamilies or sublineages. A post hoc analysis of the IS*6110* RFLP profiles of Beijing strains in this study found that 6 (2.3%) were <70% homologous to the profiles of the 19 Beijing reference strains (*33*) and may therefore represent atypical/ancient Beijing strains (*31*); these 6 isolates had nonclustered IS*6110* RFLP profiles.

In agreement with previous studies (*15*,*18*,*27*), the transmission of *M. tuberculosis* in our study was lower for older and for foreign-born persons and was unrelated to drug resistance. A deeper exploration into these transmission factors in the current study also demonstrates that these factors are independent of *M. tuberculosis* lineage, at least within the broad categories of Beijing and non-Beijing lineage strains.

TB incidence among foreign-born persons in immigrant-receiving countries has a characteristic and inverse relationship with increased time since arrival (*34*). Our findings demonstrate that this characteristic relationship is clearly evident for nonclustered cases that presumably result from the reactivation of latent TB infections acquired before immigration. Clustered Beijing and non-Beijing cases also appear to follow a similar pattern. Despite the occurrence of nearly one quarter of clustered Beijing and non-Beijing cases within the first 2 years after arrival, transmission was not associated with time since arrival, a finding that concurs with a previous study (*35*). Nevertheless, time since arrival may still have major implications for the interpopulation transmission of *M. tuberculosis* (*35*). Collectively, these findings emphasize the need for screening and prevention activities in foreign-born persons as a critical means of reducing the reactivation of latent TB infection as early after arrival as possible (*36*). It also reinforces the need for high-income countries to increase their funding of efforts to expand TB care in high incidence countries (*37*).

This study reaffirms that foreign-born persons are not a major source of *M. tuberculosis* transmission (including Beijing strains) despite their high case rates (*15*,*18*,*21*). Rather, the proportion of nonclustered cases suggests that the reactivation of latent TB infection accounts for 82% of foreign-born case-patients (i.e., 80% and 83% of foreign-born Beijing and non-Beijing case-patients, respectively). The inevitable importation of pathogens, such as Beijing strains, therefore should not be viewed so much as a threat as a challenge. The challenge lies in the host country’s resolve to prevent the reactivation of latent TB infection in recently arrived immigrants and in a larger population of aging immigrants while contending with constantly evolving immigration patterns (*34*).

The maintenance of a comprehensive provincial TB dataset derived through the amalgamation of TB Registry and mycobacteriology data was crucial for this study and the general evaluation of TB prevention and care in Alberta. The accuracy of strain classification also was enhanced through use of an unambiguous and validated genotyping method (*19*,*20*). Because Alberta is 1 of 4 primary immigrant-receiving provinces in Canada that has a similar immigration pattern as 2 of the other 3 (i.e., Ontario and British Columbia), the study results are anticipated to have national relevance. The generalizability of the study results to other low TB incidence immigrant-receiving countries will be influenced by the degree to which their immigration patterns are similar.

Unavoidable sampling limitations will have produced underestimates in clustering (*38*) and affected the transmission indices (*39*). Nevertheless, sampling bias was minimized in several ways: use of the provincial TB Registry for case identification; culture confirmation of >85% of TB cases in Alberta; availability of an expansive study period; and inclusion of 99% of eligible culture-confirmed pulmonary TB cases. Although a common practice in transmission studies, excluding nonpulmonary secondary cases could produce underestimates in clustering and transmission indices. However, sensitivity analyses in this study found the effect of this limitation to be minimal, the overall associations with transmission being unaffected by the inclusion of nonpulmonary secondary cases.

The transmission index used in this study, while advantageous for quantifying recent transmission within an expansive study period (*21*), is subject to the same limitations as other TB transmission indices (*39*). Overestimates in clustering may have resulted from the common molecular epidemiologic assumption that cases with identical DNA fingerprints were part of a transmission cluster (*4*,*27*). Although bias may have been introduced by excluding 31% of cases from the analysis of recent transmission, the effect on the study results probably is minimal because of the similarities of included and excluded cases. Last, the relatively small number of secondary Beijing cases and Beijing cases among Canadian-born persons in this study limited the ability to comprehensively assess the cross-population transmission of Beijing strains and the strain-specific transmission patterns in Canadian-born Aboriginal peoples.

This study demonstrated that Beijing strains are not independently associated with increased clustering or a larger number of secondary cases than non-Beijing strains in a setting with comprehensive and effective TB prevention and care practices (*40*). Combined with the uncommon transmission of *M. tuberculosis* by foreign-born persons in this and other studies that led to disease (*15*,*18*), there appears to be little cause for concern about the importation and subsequent transmission of Beijing strains in low TB incidence immigrant-receiving settings.

Technical Appendix Kaplan-Meier estimates of the probability of an identical DNA fingerprint pattern match in respiratory tuberculosis cases in Alberta, Canada, 1991–2007.

## References

[1] European Concerted Action on New Generation Genetic Markers and Techniques for the Epidemiology and Control of Tuberculosis. Beijing genotype *Mycobacterium tuberculosis* and drug resistance. Emerg Infect Dis. 2006;12:736–43. 10.3201/eid1205.05040016704829PMC3374453

[2] Cowley D, Govender D, February B, Wolfe M, Steyn L, Evans J, Recent and rapid emergence of W-Beijing strains of *Mycobacterium tuberculosis* in Cape Town, South Africa. Clin Infect Dis. 2008;47:1252–9. 10.1086/59257518834315

[3] Brudey K, Driscoll JR, Rigouts L, Prodinger WM, Gori A, Al-Hajoj SA, *Mycobacterium tuberculosis* complex genetic diversity: mining the fourth international spoligotyping database (SpoIDB4) for classification, population genetics and epidemiology. BMC Microbiol. 2006;6:23. 10.1186/1471-2180-6-2316519816PMC1468417

[4] Gallego B, Sintchenko V, Jelfs P, Coiera E, Gilbert GL. Three-year longitudinal study of genotypes of *Mycobacterium tuberculosis* in a low prevalence population. Pathology. 2010;42:267–72. 10.3109/0031302100363134620350221

[5] Kato-Maeda M, Kim EY, Flores L, Jarlsberg LG, Osmond D, Hopewell PC. Differences among sublineages of the East-Asian lineage of *Mycobacterium tuberculosis* in genotypic clustering. Int J Tuberc Lung Dis. 2010;14:538–44.20392345PMC3625672

[6] Langlois-Klassen D, Kunimoto D, Saunders LD, Chui L, Boffa J, Menzies D, A population-based cohort study of *Mycobacterium tuberculosis* Beijing strains: an emerging public health threat in an immigrant-receiving country? PLoS ONE. 2012;7:e38431. 10.1371/journal.pone.003843122679504PMC3367965

[7] Devaux I, Kremer K, Heersma H, Van Soolingen D. Clusters of multidrug-resistant *Mycobacterium tuberculosis* cases, Europe. Emerg Infect Dis. 2009;15:1052–60. 10.3201/eid1507.08099419624920PMC2744220

[8] Ghebremichael S, Groenheit R, Pennhag A, Koivula T, Andersson E, Bruchfeld J, Drug resistant *Mycobacterium tuberculosis* of the Beijing genotype does not spread in Sweden. PLoS ONE. 2010;5:e10893. 10.1371/journal.pone.001089320531942PMC2878347

[9] López B, Aguilar D, Orozco H, Burger M, Espitia C, Ritacco V, A marked difference in pathogenesis and immune response induced by different *Mycobacterium tuberculosis* genotypes. Clin Exp Immunol. 2003;133:30–7. 10.1046/j.1365-2249.2003.02171.x12823275PMC1808750

[10] Manca C, Reed MB, Freeman S, Mathema B, Kreiswirth B, Barry CE III, Differential monocyte activation underlies strain-specific *Mycobacterium tuberculosis* pathogenesis. Infect Immun. 2004;72:5511–4. 10.1128/IAI.72.9.5511-5514.200415322056PMC517425

[11] Toungoussova OS, Caugant DA, Sandven P, Mariandyshev AO, Bjune G. Impact of drug resistance on fitness of *Mycobacterium tuberculosis* strains of the W-Beijing genotype. FEMS Immunol Med Microbiol. 2004;42:281–90. 10.1016/j.femsim.2004.05.01215477041

[12] Aguilar D, Hanekom M, Mata D, Gey van Pittius NC, van Helden PD, Warren RM, *Mycobacterium tuberculosis* strains with the Beijing genotype demonstrate variability in virulence associated with transmission. Tuberculosis (Edinb). 2010;90:319–25. 10.1016/j.tube.2010.08.00420832364

[13] Theus S, Eisenach K, Fomukong N, Silver RF, Cave MD. Beijing family *Mycobacterium tuberculosis* strains differ in their intracellular growth in THP-1 macrophages. Int J Tuberc Lung Dis. 2007;11:1087–93.17945065

[14] Coscolla M, Gagneux S. Does *M. tuberculosis* genomic diversity explain disease diversity? Drug Discov Today Dis Mech. 2010;7:e43–59. 10.1016/j.ddmec.2010.09.00421076640PMC2976975

[15] Borgdorff MW, Behr MA, Nagelkerke NJ, Hopewell PC, Small PM. Transmission of tuberculosis in San Francisco and its association with immigration and ethnicity. Int J Tuberc Lung Dis. 2000;4:287–94.10777075

[16] World Health Organization. Global tuberculosis control: surveillance, planning, financing: WHO report 2008; 2008 [cited 2011 Apr 18]. http://www.who.int/tb/publications/global_report/2008/pdf/fullreport.pdf

[17] Public Health Agency of Canada, Canadian Lung Association/Canadian Thoracic Society. Canadian tuberculosis standards. 6th ed. Long R, Ellis E, eds. Ottawa (ON): Her Majesty the Queen in Right of Canada, represented by the Minister of Public Works and Government Services Canada; 2007.

[18] Kunimoto D, Sutherland K, Wooldrage K, Fanning A, Chui L, Manfreda J, Transmission characteristics of tuberculosis in the foreign-born and the Canadian-born populations of Alberta, Canada. Int J Tuberc Lung Dis. 2004;8:1213–20.15527153

[19] Gagneux S, DeRiemer K, Van T, Kato-Maeda M, de Jong BC, Narayanan S, Variable host-pathogen compatibility in *Mycobacterium tuberculosis.* Proc Natl Acad Sci U S A. 2006;103:2869–73. 10.1073/pnas.051124010316477032PMC1413851

[20] Tsolaki AG, Gagneux S, Pym AS, Goguet de la Salmoniere YO, Kreiswirth BN, Van Soolingen D, Genomic deletions classify the Beijing strains as a distinct genetic lineage of *Mycobacterium tuberculosis.* J Clin Microbiol. 2005;43:3185–91. 10.1128/JCM.43.7.3185-3191.200516000433PMC1169157

[21] Borgdorff MW, van den Hof S, Kremer K, Verhagen L, Kalisvaart N, Erkens C, Progress towards tuberculosis elimination: secular trend, immigration and transmission. Eur Respir J. 2010;36:339–47. 10.1183/09031936.0015540919996188

[22] Jasmer RM, Hahn JA, Small PM, Daley CL, Behr MA, Moss AR, A molecular epidemiologic analysis of tuberculosis trends in San Francisco, 1991–1997. Ann Intern Med. 1999;130:971–8. 10.7326/0003-4819-130-12-199906150-0000410383367

[23] Hosmer DW, Lemeshow S. Applied logistic regression. 2nd ed. New York: Wiley; 2000.

[24] Frieden TR, Sherman LF, Maw KL, Fujiwara PI, Crawford JT, Nivin B, A multi-institutional outbreak of highly drug-resistant tuberculosis: epidemiology and clinical outcomes. JAMA. 1996;276:1229–35. 10.1001/jama.1996.035401500310278849750

[25] de Jong BC, Hill PC, Aiken A, Awine T, Antonio M, Adetifa IM, Progression to active tuberculosis, but not transmission, varies by *Mycobacterium tuberculosis* lineage in The Gambia. J Infect Dis. 2008;198:1037–43. 10.1086/59150418702608PMC2597014

[26] Marais BJ, Hesseling AC, Schaaf HS, Gie RP, Van Helden PD, Warren RM. *Mycobacterium tuberculosis* transmission is not related to household genotype in a setting of high endemicity. J Clin Microbiol. 2009;47:1338–43. 10.1128/JCM.02490-0819261801PMC2681880

[27] Nava-Aguilera E, Andersson N, Harris E, Mitchell S, Hamel C, Shea B, Risk factors associated with recent transmission of tuberculosis: systematic review and meta-analysis. Int J Tuberc Lung Dis. 2009;13:17–26.19105874

[28] Lohmann EM, Koster BFPJ, le Cessie S, Kamst-van Agterveld MP, van Soolingen D, Arend SM. Grading of a positive sputum smear and the risk of *Mycobacterium tuberculosis* transmission. Int J Tuberc Lung Dis. 2012;16:1477–84. 10.5588/ijtld.12.012922964038

[29] Borgdorff MW, van Deutekom H, de Haas PE, Kremer K, van Soolingen D. *Mycobacterium tuberculosis,* Beijing genotype strains not associated with radiological presentation of pulmonary tuberculosis. Tuberculosis (Edinb). 2004;84:337–40. 10.1016/j.tube.2003.10.00215207809

[30] Portevin D, Gagneux S, Comas I, Young D. Human macrophage responses to clinical isolates from the *Mycobacterium tuberculosis* complex discriminate between ancient and modern lineages. PLoS Pathog. 2011;7:e1001307. 10.1371/journal.ppat.100130721408618PMC3048359

[31] Mokrousov I, Narvskaya O, Otten T, Vyazovaya A, Limeschenko E, Steklova L, Phylogenetic reconstruction within *Mycobacterium tuberculosis* Beijing genotype in northwestern Russia. Res Microbiol. 2002;153:629–37. 10.1016/S0923-2508(02)01374-812558181

[32] Tsao TCY, Hong J, Li LF, Hsieh MJ, Liao SK, Chang KSS. Imbalances between tumor necrosis factor-alpha and its soluble receptor forms, and interleukin-1 beta and interleukin-1 receptor antagonist in BAL fluid of cavitary pulmonary tuberculosis. Chest. 2000;117:103–9. 10.1378/chest.117.1.10310631206

[33] Kremer K, Glynn JR, Lillebaek T, Niemann S, Kurepina NE, Kreiswirth BN, Definition of the Beijing/W lineage of *Mycobacterium tuberculosis* on the basis of genetic markers. J Clin Microbiol. 2004;42:4040–9. 10.1128/JCM.42.9.4040-4049.200415364987PMC516354

[34] Langlois-Klassen D, Wooldrage K, Manfreda J, Sutherland K, Ellis E, Phypers M, Piecing the puzzle together: foreign-born tuberculosis in an immigrant-receiving country. Eur Respir J. 2011;38:895–902. 10.1183/09031936.0019661021436350

[35] Vanhomwegen J, Kwara A, Martin M, Gillani FS, Fontanet A, Mutungi P, Impact of immigration on the molecular epidemiology of tuberculosis in Rhode Island. J Clin Microbiol. 2011;49:834–44. 10.1128/JCM.01952-1021159930PMC3067685

[36] Smieja MJ, Marchetti CA, Cook DJ, Smaill FM. Isoniazid for preventing tuberculosis in non-HIV infected persons. Cochrane Database Syst Rev. 2000; (2):CD001363.1079664210.1002/14651858.CD001363PMC6532737

[37] Schwartzman K, Oxlade O, Barr RG, Grimard F, Acosta I, Baez J, Domestic returns from investment in the control of tuberculosis in other countries. N Engl J Med. 2005;353:1008–20. 10.1056/NEJMsa04319416148286

[38] Glynn JR, Bauer J, de Boer AS, Borgdorff MW, Fine PE, Godfrey-Faussett P, European Concerted Action on Molecular Epidemiology and Control of Tuberculosis. Interpreting DNA fingerprint clusters of *Mycobacterium tuberculosis.* Int J Tuberc Lung Dis. 1999;3:1055–60.10599007

[39] Tanaka MM, Phong R, Francis AR. An evaluation of indices for quantifying tuberculosis transmission using genotypes of pathogen isolates. BMC Infect Dis. 2006;6:92. 10.1186/1471-2334-6-9216756684PMC1538606

[40] Jensen M, Lau A, Langlois-Klassen D, Boffa J, Manfreda J, Long R. A population-based study of tuberculosis epidemiology and innovative service delivery in Canada. Int J Tuberc Lung Dis. 2012;16:43–9. 10.5588/ijtld.11.037422236844

# Research article: Transmission of *Mycobacterium tuberculosis* Beijing Strains, Alberta, Canada, 1991–2007

**Authors:** Deanne Langlois-Klassen, Ambikaipakan Senthilselvan, Linda Chui, Dennis Kunimoto, L. Duncan Saunders, Dick Menzies, Richard Long

**Affiliations:** University of Alberta, Edmonton, Alberta, Canada (D. Langlois-Klassen, A. Senthilselvan, L. Chui, D. Kunimoto, L.D. Saunders, R. Long);; Provincial Laboratory for Public Health, Edmonton (L. Chui); McGill University Health Centre, Montreal, Quebec, Canada (D. Menzies);; McGill University, Montreal (D. Menzies)

## Medscape CME Quiz

## Earning CME Credit

To obtain credit, you should first read the journal article. After reading the article, you should be able to answer the following, related, multiple-choice questions. To complete the questions (with a minimum 70% passing score) and earn continuing medical education (CME) credit, please go to www.medscape.org/journal/eid. Credit cannot be obtained for tests completed on paper, although you may use the worksheet below to keep a record of your answers. You must be a registered user on Medscape.org. If you are not registered on Medscape.org, please click on the New Users: Free Registration link on the left hand side of the website to register. Only one answer is correct for each question. Once you successfully answer all post-test questions you will be able to view and/or print your certificate. For questions regarding the content of this activity, contact the accredited provider, CME@medscape.net. For technical assistance, contact CME@webmd.net. American Medical Association’s Physician’s Recognition Award (AMA PRA) credits are accepted in the US as evidence of participation in CME activities. For further information on this award, please refer to http://www.ama-assn.org/ama/pub/category/2922.html. The AMA has determined that physicians not licensed in the US who participate in this CME activity are eligible for *AMA PRA Category 1 Credits*™. Through agreements that the AMA has made with agencies in some countries, AMA PRA credit may be acceptable as evidence of participation in CME activities. If you are not licensed in the US, please complete the questions online, print the certificate and present it to your national medical association for review.

## Article Title:  Transmission of *Mycobacterium tuberculosis* Beijing Strains,  Alberta, Canada, 1991–2007

## CME Questions

1. You are seeing family of 5 who emigrated from China one year ago. The father of the family was recently diagnosed with pulmonary tuberculosis (TB), and you are worried about the potential spread of the infection, particularly with virulent strains of *Mycobacterium tuberculosis*. What should you consider in general regarding the epidemiology of the Beijing lineage of *M. tuberculosis*?

A. It accounts for 13% of all TB strains globally

B. It accounts for less than 2% of cases of TB in immigrant-receiving countries with low rates of infection

C. It is rarely resistant to treatment

D. The Beijing strain is less virulent compared with other TB strains in animal models

**2. Which of the following characteristics was most associated with TB infection with the Beijing strains in the current study?**

A. Higher proportion of foreign-born patients

B. Younger age

C. More likely to have a positive sputum smear

D. Higher rates of drug resistance

**3. What should you consider regarding the transmission of Beijing vs non-Beijing strains of TB in the current study?**

A. The Beijing strains were associated with higher rates of clustered cases

B. The Beijing strains were associated with similar rates of clustered cases compared with non-Beijing strains

C. The Beijing cases were only more likely to be clustered among foreign-born patients

D. The Beijing cases were only more likely to be clustered among drug-resistant strains

**4. Which of the following factors was most positively associated with a higher rate of secondary cases of TB in the current study?**

A. Beijing lineage

B. Age over 64 years in the index case

C. Positive sputum smear in the index case

D. Foreign-born patient in the index case

## Activity Evaluation

**1:** The activity supported the learning objectives.

Strongly Disagree				Strongly Agree
1	2	3	4	5
**2. The material was organized clearly for learning to occur.**				
Strongly Disagree				Strongly Agree
1	2	3	4	5
**3. The content learned from this activity will impact my practice.**				
Strongly Disagree				Strongly Agree
1	2	3	4	5
**4. The activity was presented objectively and free of commercial bias.**				
Strongly Disagree				Strongly Agree
1	2	3	4	5
